# Optical coherence tomography angiography for the diagnosis of granulomatosis with polyangiitis with serous retinal detachment: A case report

**DOI:** 10.1097/MD.0000000000024789

**Published:** 2021-02-19

**Authors:** Noriko Takashi, Aya Nakamura, Keiko Kataoka, Yoshihiko Usui, Yasuki Ito, Hiroki Kaneko

**Affiliations:** a Department of Ophthalmology, Nagoya University Graduate School of Medicine; b Department of Ophthalmology, Nagoya Medical Center, Nagoya; c Department of Ophthalmology, Tsushima Municipal Hospital, Tsushima; d Department of Ophthalmology, Tokyo Medical University, Tokyo, Japan.

**Keywords:** central serous chorioretinopathy, choroidal neovascularization, granulomatosis with polyangiitis, optical coherence tomography angiography

## Abstract

**Rationale:**

Granulomatosis with polyangiitis (GPA) is a rare systemic autoimmune disease of unknown etiology. GPA affects multiple ocular tissues, most commonly the orbit, conjunctiva, cornea, and sclera. Retinal and choroidal manifestations are rare in GPA, but they often include choroidal neovascularization (CNV).

**Patient concerns:**

A 36-year-old man was diagnosed with GPA. He had been taking oral steroid treatment for 8 years. He experienced disease recurrence and the dose of oral prednisolone was increased after steroid pulse therapy. Fundus examination showed small retinal pigment epithelial detachment and serous retinal detachment (SRD). Optical coherence tomography (OCT) revealed a protruded lesion inside the SRD. Fluorescein angiography (FA) showed a small, dot-shaped fluorescein leakage in the SRD, and indocyanine green fluorescein fundus angiography showed choroidal vascular hyperpermeability that was consistent with the hyperfluorescence seen with FA. We had to determine whether the protruded lesion inside the SRD was CNV secondary to the inflammation due to GPA or whether it was central serous chorioretinopathy (CSC)-like condition caused by high-dose steroid treatment.

**Diagnoses:**

We confirmed that the SRD was due to CSC but not CNV because the protruded lesion examined by B-scan OCT angiography (OCTA) showed no blood flow.

**Interventions:**

We decided to reduce the dose of steroid.

**Outcomes:**

Since the reduction of steroids, no sign of worsening in the protruded lesions with SRD has been observed.

**Lessons:**

We therefore propose the effectiveness of this advanced function of OCTA for the examination of blood flow signal images to detect CNV.

## 1 Introduction

Granulomatosis with polyangiitis (GPA), formerly known as Wegener's granulomatosis, is a rare systemic autoimmune disease with an unknown etiology. The prevalence of GPA is estimated to be 3 per 100,000, and the annual incidence is approximately 8 to 10 per 1 million, but it varies depending on a person's ethnicity and geographic location.^[1]^ GPA presents with a wide range of clinical manifestations of varying severity. GPA also affects multiple ocular tissues, most commonly the orbit, conjunctiva, cornea, and sclera.^[2]^ Retinal and choroidal manifestations are rare in GPA, but they are recognized as inflammation-based changes, such as retinitis, chorioretinitis, macular edema, exudative retinal detachment, and retinal necrosis. Choroidal inflammation due to GPA can also induce choroidal neovascularization (CNV).^[3]^ Here, we report a case of retinal and choroidal manifestation in a patient with GPA and propose the effectiveness of examination with the advanced B-scan image function of optical coherence tomography angiography (OCTA) to detect blood flow signals.

## 2 Case report

**Figure 1:**
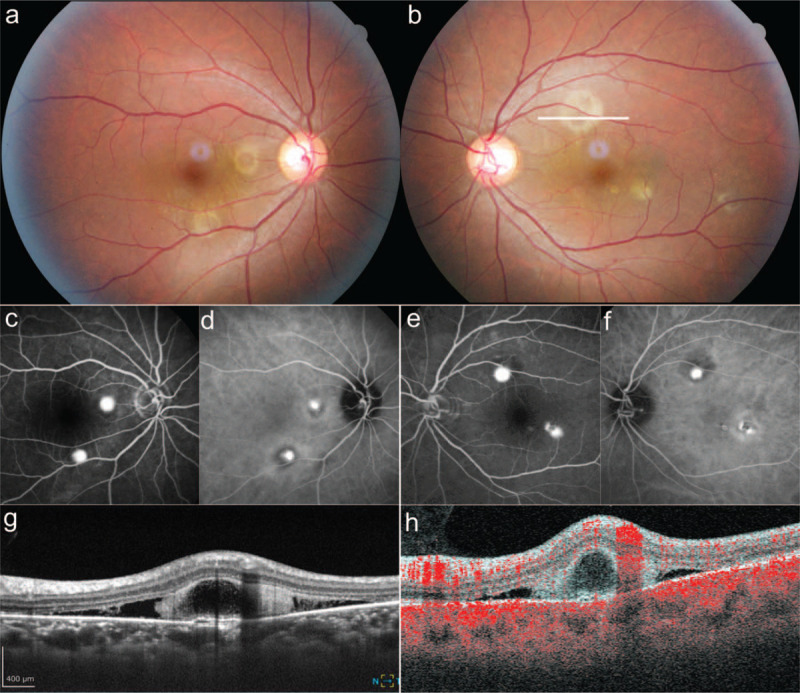
Images from a patient with granulomatosis with polyangiitis. **(a,b)** Color fundus images. Multiple yellow-white lesions in both eyes are noted. (a: right, b: left) **(c, d)**: FA (c) and ICGA (d) images from the right eye. Hyperfluorescent leakages consistent with the yellow-white lesions are noted. **(e, f)** FA (e) and ICGA (f) images from the left eye. Hyperfluorescent leakages consistent with the yellow-white lesions are noted. **(g)** OCT image from a yellow-white lesion in the left eye (white line in [b]). hyperreflective protrusion in the serous retinal detachment is noted. **(h)** B-scan OCT angiography from the same protruded lesion as that in (g). Note that there is no blood flow signal (colored in red) inside the protruded lesion, indicating that it is not an active choroidal neovascularization. FA = fluorescein angiography, ICGA = indocyanine green angiography, OCT = optical coherence tomography, OCTA = OCT-angiography.

A 36-year-old man was diagnosed with GPA in 2011. He has been followed up by private practice physicians and his clinical condition was well-controlled with oral prednisolone (0.075 mg/kg) and mizoribine (100 mg) for 8 years. In early 2019, he was referred to our hospital because he experienced disease recurrence with symptoms of arthritis and rapid deterioration of renal function. In addition, he underwent partial small bowel resection for small bowel bleeding. His renal function did not improve, and he was placed on maintenance dialysis. He was given 0.15 mg/kg oral prednisolone after steroid pulse therapy (1,000 mg/d for 3 days). In July 2019, he was referred to the ophthalmology department for the evaluation of GPA and steroid-induced ocular complications. His visual acuity was 20/20 in both eyes (OU). Intraocular pressures were 18 mm Hg in the right eye (OD) and 15 mm Hg in the left eye (OS). Results of slit lamp examination of the anterior segment were unremarkable. Fundus examination and optical coherence tomography (OCT) revealed a small retinal pigment epithelial detachment (PED) in the major temporal arcade in the OD and a small PED and serous retinal detachment (SRD) superior to the optic nerve head in the OS. The patient had previously discontinued visiting the ophthalmology department after 2011 because he had not had any subjective ocular symptoms. However, in January 2019, he presented to the ophthalmology department again with subjective symptoms of vision deterioration. His visual acuity was 20/20 in the OD and 20/25 in the OS. IOPs were 14 mm Hg in the OU. Results of a fundus examination showed multiple small circular SRDs in the major temporal arcade in OU. OCT detected hyperreflective protrusion that had hyporeflective bubble-like structure inside hyperreflective materials in the SRD. In addition, the choroid under the hyperreflective protrusions contained pachyvessel and low serous PED. Fluorescein angiography (FA) showed small, dot-shaped hyperfluorescent leakage from the SRDs. Indocyanine green angiography (ICGA) showed choroidal vascular hyperpermeability that was consistent with the hyperfluorescence seen with FA. To determine the appropriate treatment, it was necessary to determine whether the protruded lesions contained choroidal neovascularization (CNV) secondary to the inflammation due to GPA. Alternatively, the protruded lesions could have been fibrin accumulations and the SRDs could have been caused by central serous chorioretinopathy (CSC), which in turn was probably caused by the high-dose steroid treatment. We employed B-scan OCT angiography (OCTA) to detect blood flow. We identified that the protruded lesions had no blood flow inside of them, confirming that the diagnosed SRDs were CSC but not CNV. We decided to reduce the dose of steroid (Fig. 1). Since the reduction of steroids, no sign of worsening in the protruded lesions with SRD has been observed for 1.5 years.

## 3 Discussion

Retinal and choroidal manifestations have been reported in GPA; however, their prevalence is not higher than that of other ocular manifestations. Retinal and choroidal manifestations in GPA include SRD and CNV, and they are mainly caused by severe inflammation from GPA. For CNV associated with posterior uveitis, immunosuppressive treatment with steroid is reportedly effective.^[4]^ Nevertheless, steroid treatment is thought to induce CSC.^[5]^ Sometimes, even after examining FA and IA results, it is very difficult to determine whether SRD and/or PED are caused by the uncontrolled inflammation of posterior uveitis or are the side effects of steroid treatment for posterior uveitis. As we showed here, for a case with hyperreflective protrusions in SRD, it is particularly important to evaluate precisely the existence of CNV, a vision-threatening disease. For the last few decades, CNV has been diagnosed only by FA and ICGA. However, Fujita et al recently showed that the accuracy and the diagnosis rate of PCV by IA is not significantly different from those of blood flow examination by B-scan OCTA.^[6]^ Especially for patients with renal dysfunction, multiple examinations with systemic infusion of dyes is not preferable. In the present case, both FA/ICGA and OCTA were performed, and OCTA confirmed the absence of an internal blood flow signal in the protruded lesion, which led us to exclude the possibility of CNV.

Diagnosis of CNV secondary to posterior uveitis or CSC caused by steroid treatment for posterior uveitis is critical because misjudgment might cause exacerbation of the disease. The existence or absence of a blood flow signal, as measured by B-scan OCTA, is very informative and useful for confirming the diagnosis of ocular inflammatory diseases.

## 4 Ethical statement

Written informed consent was obtained from the patient. Ethical approval was obtained from the Ethics Committee of the Nagoya University Hospital, Nagoya, Japan, in accordance with the ethical guidelines of the 1975 Declaration of Helsinki.

## Acknowledgment

The authors thank Editage (www.editage.com) for English language editing.

## Author contributions

**Conceptualization:** Noriko Takashi, Aya Nakamura.

**Resources:** Noriko Takashi, Aya Nakamura.

**Supervision:** Yoshihiko Usui, Keiko Kataoka, Yasuki Ito.

**Writing – original draft:** Aya Nakamura, Hiroki Kaneko.

**Writing – review & editing:** Hiroki Kaneko.
